# Supratherapeutic drug concentration triggers: A novel data‐driven approach to assess their value for medication safety surveillance in intensive care

**DOI:** 10.1002/bcp.70610

**Published:** 2026-05-21

**Authors:** Anne Paulien Langermans, Izak A. R. Yasrebi‐de Kom, Dylan Wayne de Lange, Nicolette Fransisca de Keizer, Joanna E. Klopotowska, RESCUE Study group, C. S. C. Bouman, E. N. van Roon, P. F. Schutte, J. ten Cate, D. van Balen, S. Hendriks, C. Lau, W. J. Vermeijden, A. Beishuizen, J. B. Masselink, P. E. Spronk, H. J. M. van Kan, P. W. de Feiter, E. J. Wils, A. Wieringa, A. J. Valkenburg, W. M. van den Bergh, M. H. Renes, W. Bult, E. de Jonge, M. Hoeksema, E. J. Wesselink, I. M. Purmer, B. E. Bosma, S. H. W. van Bree, H. J. W. Lammers, R. J. Bosman, E. J. F. Franssen, A. Karakus, M. Sigtermans, E. M. Kuck, D. A. Dongelmans, M. J. H. Aries, E. V. Uijtendaal, M. C. Reuland

**Affiliations:** ^1^ Department of Medical Informatics, Amsterdam Public Health research institute Amsterdam University Medical Centre Amsterdam The Netherlands; ^2^ ChipSoft Amsterdam The Netherlands; ^3^ Department of Intensive Care and Dutch Poison Information Centre University Medical Centre Utrecht Utrecht The Netherlands; ^4^ Department of Intensive Care Medicine Amsterdam UMC location University of Amsterdam Amsterdam The Netherlands; ^5^ Department of Clinical Pharmacy and Pharmacology Frisius medical Center Leeuwarden The Netherlands; ^6^ Department of Intensive Care The Netherlands Cancer Institute Amsterdam The Netherlands; ^7^ Department of Pharmacy & Pharmacology The Netherlands Cancer Institute Amsterdam The Netherlands; ^8^ Department of Intensive Care Albert Schweitzer Ziekenhuis Dordrecht The Netherlands; ^9^ Department of Hospital Pharmacy Albert Schweitzer Ziekenhuis Dordrecht The Netherlands; ^10^ Department of Intensive Care Medisch Spectrum Twente Enschede The Netherlands; ^11^ Department of Clinical Pharmacy Medisch Spectrum Twente Enschede The Netherlands; ^12^ Department of Intensive Care Medicine Gelre Hospitals Apeldoorn The Netherlands; ^13^ Department of Clinical Pharmacy Gelre Hospitals Apeldoorn The Netherlands; ^14^ Department of Intensive Care Franciscus Gasthuis & Vlietland Rotterdam The Netherlands; ^15^ Department of Clinical Pharmacy Isala Hospital Zwolle The Netherlands; ^16^ Department of Intensive Care Isala Hospital Zwolle The Netherlands; ^17^ Department of Critical Care University Medical Center Groningen, University of Groningen Groningen The Netherlands; ^18^ Department of Clinical Pharmacy and Pharmacology University Medical Center Groningen, University of Groningen Groningen The Netherlands; ^19^ Department of Intensive Care Leiden University Medical Center Leiden The Netherlands; ^20^ Department of Anesthesiology, Intensive Care and Painmanagement Zaans Medisch Centrum Zaandam The Netherlands; ^21^ Department of Clinical Pharmacy Zaans Medisch Centrum Zaandam The Netherlands; ^22^ Department of Intensive Care Haga Hospital The Hague The Netherlands; ^23^ Department of Hospital Pharmacy Haga Hospital The Hague The Netherlands; ^24^ Department of Intensive Care Ziekenhuis Gelderse Vallei Ede The Netherlands; ^25^ Department of Hospital Pharmacy Ziekenhuis Gelderse Vallei Ede The Netherlands; ^26^ Department of Intensive Care Onze Lieve Vrouwe Gasthuis Amsterdam The Netherlands; ^27^ Department of Clinical Pharmacy Onze Lieve Vrouwe Gasthuis Amsterdam The Netherlands; ^28^ Department of Intensive Care Diakonessenhuis Utrecht Utrecht The Netherlands; ^29^ Department of Hospital Pharmacy Diakonessenhuis Utrecht Utrecht The Netherlands; ^30^ Department of Intensive Care Maastricht University Medical Center Maastricht The Netherlands; ^31^ Mental Health and Neurosciences Institute University Maastricht Maastricht The Netherlands; ^32^ Department of Clinical Pharmacy University Medical Center Utrecht and Utrecht University Utrecht The Netherlands

**Keywords:** adverse drug events, electronic triggers, intensive care, medication safety, patient outcomes

## Abstract

**Aims:**

Electronic triggers (e‐triggers) are used as screening signals to detect potential adverse drug events (ADEs) and offer an effective system level approach for medication safety surveillance. Their clinical utility is typically evaluated through time‐consuming manual chart review by experts, limiting implementation. Estimating associations between e‐triggers and relevant ICU patient outcomes may provide a rapid, data‐driven alternative. In this study, electronic supratherapeutic drug concentration triggers (eSDCT) were used as a use case.

**Methods:**

Pseudonymized Electronic Health Record (EHR) data from adults admitted to 14 Dutch ICUs (2010–2020) were analysed. Patients with ≥1 therapeutic drug monitoring measurements were included. eSDCTs were selected based on literature, data availability and expert input. Associations with ICU Length of Stay (LOS), ICU mortality and hospital mortality were assessed using adjusted linear and logistic mixed‐effects models.

**Results:**

Among 6006 ICU admissions, 49.5% (*n* = 2973) had ≥1 of the five selected group‐level eSDCTs. Exposed admissions showed higher hospital mortality (38.2% *vs*. 27.9%), ICU mortality (30.6% *vs*. 20.3%) and longer ICU LOS (median 13.8 [IQR 5.6–28.1] *vs*. 7.5 [3.2–15.7] days) compared with unexposed admissions. After adjustment, antibiotic and immunosuppressive eSDCTs remained associated with increased ICU mortality (OR 1.47 [1.26–1.70] and 2.86 [1.56–5.24]), hospital mortality (OR 1.46 [1.27–1.68] and 1.81 [1.07–3.10]) and prolonged ICU LOS (expB 1.82 [1.71–1.94] and 2.99 [2.46–3.62]).

**Conclusion:**

Our data‐driven approach shows that antibiotic and immunosuppressive eSDCTs are associated with worse ICU patient outcomes, supporting their implementation for ICU‐level medication safety surveillance.

**What is already known about this subject**

- Electronic triggers (e‐triggers) for medication safety surveillance in the intensive care units (ICUs), improve detection of adverse drug events (ADEs).
- However, validation of these triggers typically relies on manual patient chart review by medical experts, to determine their positive predictive value, which is labour‐intensive and hampers implementation.

**What this study adds**

- A novel data‐driven approach to evaluate the clinical utility of e‐triggers by examining their association with ICU patient outcomes, using electronic supratherapeutic drug concentration triggers (eSDCTs) as a use case.
- Antibiotic and immunosuppressive eSDCTs are associated with worse ICU patient outcomes.
- These eSDCTs may support ICU‐level medication safety surveillance in the ICU.

## 1 INTRODUCTION

Adverse drug events (ADEs) are common in patients admitted into intensive care units (ICU) and are associated with increased mortality rates, longer ICU length of stay (ICU LOS) and increased healthcare costs.^1^, ^2^ The incidence of ADEs in ICU setting is relatively high compared with other hospital departments and ranges from 10 to 106 per 1000 patient days.^3^, ^4^, ^5^, ^6^, ^7^ This may be due to frequent polypharmacy, use of high‐risk drugs and organ function impairment affecting pharmacokinetics and pharmacodynamics.^1^, ^7^ Roughly 20% to 68% of the ADEs in the ICU are deemed preventable as they stem from medication errors.^6^, ^8^ Approximately 20% of these preventable ADEs can be attributed to dosing errors, such as overdosing, leading to supratherapeutic drug concentrations.^6^

To monitor and improve medication safety in ICU, it is important to track ADEs to identify areas for improvement.^9^, ^10^ The standard practice to do so is voluntary reporting.^11^ However, it has been estimated that less than 5% of all ADEs are reported this way.^10^ An alternative or complementary approach is the use of the so‐called electronic triggers (e‐triggers). E‐triggers are signals (e.g., supratherapeutic drug concentrations, ordering antidotes and ordering an electrocardiogram) identified in electronic health records (EHRs) that may indicate potential ADEs.^4^, ^12^, ^13^ Use of e‐triggers has shown to be more time‐efficient and results in a substantially higher ADE yield and broader ADE type capture.^14^, ^15^ Implementing ADE e‐triggers on a hospital‐ or department‐level dashboard, supports system level medication safety surveillance.^14^

However, before e‐triggers can be implemented for such a purpose, their clinical utility needs to be established. This has traditionally relied on a manual chart review by experts to determine the positive predictive value (PPV) of candidate e‐triggers, with higher PPVs indicating more efficient identification of actual ADEs.^9^, ^16^, ^17^ Yet, given the time‐consuming nature of manual patient chart reviews by experts, such evaluations are challenging, hampering the use of e‐triggers for medication safety surveillance.

A novel data‐driven alternative for evaluating the clinical utility of e‐triggers may be assessing the associations of e‐triggers with ICU patient outcomes. A strong association does not imply causation but may indicate that an e‐trigger is linked to clinically important ICU patient outcomes and therefore holds value for system level medication safety surveillance and quality of care improvement initiatives. To the best of our knowledge, no previous studies in the ICU setting have investigated a data‐driven approach to determine the clinical utility of ADE e‐triggers by estimating their associations with ICU LOS, ICU mortality and hospital mortality, while adjusting for confounding factors.

Therefore, the objective of this study was to evaluate the potential of such an approach as an alternative to manual patient chart review by medical experts. To test this, electronic supratherapeutic drug concentration triggers (eSDCTs) served as use cases. As mentioned earlier, preventable ADEs due to overdose are common in the ICU, and eSDCTs could support in detecting such ADEs.^5^, ^8^

## 2 METHODS

This study is reported according to the REporting of studies Conducted using Observational Routinely‐collected health Data for PharmacoEpidemiology (RECORD‐PE) guideline (see Supporting Information S1 for the RECORD‐PE list).^18^ This study was part of the study “Towards a leaRning mEdication Safety system in a national network of intensive Care Units—timely detection of adverse drug Events” (RESCUE).

### 2.1 Study design

This multicentre observational study reused routinely collected pseudonymized data from EHR systems of 14 ICUs in the Netherlands, registered between 01‐01‐2010 and 01‐01‐2020. The RESCUE project was exempted from requiring ethics approval (waiver W19_433 # 19.499) on 14 November 2019 by the Medical Ethics Committee of the Amsterdam University Medical Centers, location University of Amsterdam, The Netherlands, as it did not fall within the scope of the Dutch Medical Research Involving Human Subjects Act (WMO).

### 2.2 Data collection

The data consisted of two linked pseudonymized datasets: (1) the Minimal Data Set (MDS) of the National Intensive Care Evaluation (NICE) quality registry, which includes among others demographics, acute physiology measurements, ICU patient outcomes, reasons for ICU admission and (chronic and acute) comorbidities^19^ and (2) therapeutic drug monitoring (TDM) measurements separately extracted from the EHRs of the 14 ICUs. These two datasets were linked via the unique NICE admission number available in both datasets.

### 2.3 Selection of electronic supratherapeutic drug concentration triggers

We selected eSDCTs in three steps: a literature review, inspection of the available TDM measurements in the TDM dataset and ICU expert consultation. The literature review was conducted to identify previously investigated eSDCTs in the ICU setting, including their threshold values. Inclusion criteria were defined prior to the literature search. We included original studies written in English or Dutch, which described the development and evaluation (e.g., through calculating a PPV) of an eSDCT for the detection of (potential) ADEs in adult ICU patients. No restrictions were applied on the timeframe in which studies had to be published. We subsequently searched the MEDLINE database using the search strategy presented in Supporting Information S2. Next, we inspected the TDM dataset for the availability of any TDM measurement to identify for which drugs TDM was conducted.

Lastly, a list of eSDCTs from the literature review and eSDCT for drugs for which at least 500 TDM measurements were available in the TDM dataset, were presented to ICU experts. Experts were practicing ICU physicians or hospital pharmacists with ICU specialty. The experts assessed which eSDCT they would consider for ICU‐level medication safety surveillance by elaborating on the ADE potential of the respective drug in situations where a supratherapeutic concentration occurs in an ICU patient. If ADE potential was considered high by at least half of the experts, the eSDCT was selected for further investigation in this study.

The literature review was also used to identify suitable supratherapeutic and subtherapeutic thresholds for the selected eSDCT. To align with Dutch clinical practice, the final thresholds for the selected eSDCTs, were aligned with the Dutch standard for TDM.^20^

### 2.4 Exposures to selected eSDCTs

An exposed ICU admission was defined as an admission that met the conditions specified in the eSDCT. These conditions were: *IF* a patient received [the drug under investigation] AND the concentration of [the drug under investigation] was measured AND a patient was exposed to a supratherapeutic concentration of [the drug under investigation] *THEN* flag the admission as exposed. An unexposed admission was defined as an admission that met the following conditions: *IF* a patient received [the drug under investigation] AND the concentration of [the drug under investigation] was measured AND a patient was not exposed to a supratherapeutic concentration of [the drug under investigation] *THEN* flag the admission as unexposed.

### 2.5 Outcome variables

We investigated three patient‐centred outcomes in the ICU context: ICU LOS, ICU mortality and hospital mortality. ICU LOS was calculated as fractional days using the exact date and time of ICU admission and discharge. Patients who died during their ICU stay were registered as deaths in the ICU. Hospital mortality was defined as patients who died during their ICU stay or after being transferred to another department in the same hospital.

### 2.6 ICU admission inclusion

ICU admissions were included if the patient was 18 years or older at ICU admission. We stratified the cohort by including ICU admissions with at least one TDM measurement for a drug listed in an eSDCT. Admissions with missing data for one or more of the patient outcomes were excluded. For patients with multiple ICU admissions, only the first ICU admission within the same hospitalization was used. Readmissions were not included.

### 2.7 Statistical analysis

Characteristics of the included ICU admissions were obtained through counts with percentages or medians with interquartile ranges (IQRs) as appropriate. Missing data were imputed using the multivariate imputation by chained equations (mice) R package. Continuous variables were imputed using predictive mean matching, and categorical variables were imputed using random sampling from the nonmissing values (more information can be found in Supporting Information S3). We estimated the associations between the presence of an eSDCT and the three patient outcomes, where the LOS outcome was log‐transformed to meet the normality assumption, through linear (for LOS) and logistic (for ICU and hospital mortality) mixed‐effect regression models. A random intercept for ICU was used to address the potential clustering of data within ICUs, unless eSDCT‐specific sample size did not allow for this due to low admission counts per ICU.

For each of the three patient outcomes, we fitted adjusted models, accounting for potential confounders. The potential confounders were identified through literature review. ICU and hospital mortality were corrected for body mass index (BMI), APACHE IV probability, chronic cardiovascular and renal insufficiency, confirmed infection, cardiopulmonary resuscitation (CPR), cerebrovascular accident (CVA) and subtherapeutic drug concentration levels. ICU LOS was corrected for age, BMI, Glasgow coma scale, confirmed infection, chronic renal and cardiovascular insufficiency, CPR, CVA, dysrhythmia, chronic obstructive pulmonary disease and subtherapeutic drug concentration levels. We hypothesized that subtherapeutic drug concentrations may indicate potential therapy failure and could therefore be associated with prolonged ICU LOS as well as ICU and hospital mortality. For this reason, subtherapeutic concentrations were included as a confounder in all models. We excluded dichotomous categorical variables with a prevalence below 1% to ensure a proper model fit while minimizing the risk of residual confounding.

The estimates of the linear models were reported as exponentiated coefficient *β* (exp(*β*)) with 95% confidence intervals (CIs). The exp(*β*) represents the multiplicative effect on the mean LOS. The estimates of the logistic regression models were reported as odds ratios (ORs), along with 95% CIs. All analyses were conducted in R version 4.4.3, and we used the lme4 R package for mixed‐effect modelling.^21^, ^22^

## 3 RESULTS

### 3.1 Selection of electronic supratherapeutic drug concentration triggers

The literature review resulted in three studies from which we selected five drugs potentially suitable for an eSDCT: vancomycin, valproic acid, phenytoin, digoxin and quinidine (see Supporting Information S4).^7^, ^23^, ^24^ A total of 13 drugs met the inclusion criterion of at least 500 available TDM measurements. Within these 13 drugs, all drugs identified via literature review were present except for quinidine due to few TDM measurements. The 13 drugs were subsequently presented to the expert panel in form of eSDCTs. The panel consisted of six experts: three ICU physicians and three hospital pharmacists. They were affiliated with three academic hospitals (more information is provided in Supporting Information S5). Five of the 13 drugs were excluded based on either having low ADE potential (acyclovir and ganciclovir), TDM conducted due to intoxication (paracetamol), expected to be replaced by an alternative drug in the near future (gentamicin) or because TDM is mainly conducted for research purposes and not routine care (amphotericin B). The final eSDCTs selection included eight drugs, aggregated into five drug‐group eSDCTs (Table 1). Drugs were grouped because TDM measurements for individual drugs were limited, thereby necessitating analyses at the drug group level.

**TABLE 1:** Included drug groups with their corresponding drugs and supratherapeutic drug concentration thresholds.

Drug	Type of threshold	Total number of TDM measurements	Supratherapeutic drug concentration threshold
**Antibiotics**			
Flucloxacillin	Trough concentration	965	>125 mg/L
Tobramycin	Trough concentration	1810	>1 mg/L
Tobramycin	Peak concentration	89	>20 mg/L
Vancomycin	Continuous administration	885	>25 mg/L
Vancomycin	Trough concentration	28 230	>20 mg/L
Vancomycin	Peak concentration	309	>40 mg/L
**Cardiac glycosides**			
Digoxin	Trough concentration	782	>2 μg/L
**Immunosuppressives**			
Tacrolimus	Trough concentration	6777	>20 μg/L
**Antiepileptics**			
Phenytoin	Trough concentration	841	>20 mg/L
Phenytoin	Free fraction concentration	171	>2 mg/L
Valproic acid	Trough concentration	878	>120 mg/L
**Antifungals**			
Voriconazole	Trough concentration	1096	>6 mg/L

Abbreviation: TDM, therapeutic drug monitoring.

### 3.2 Admission inclusion

**FIGURE 1:**
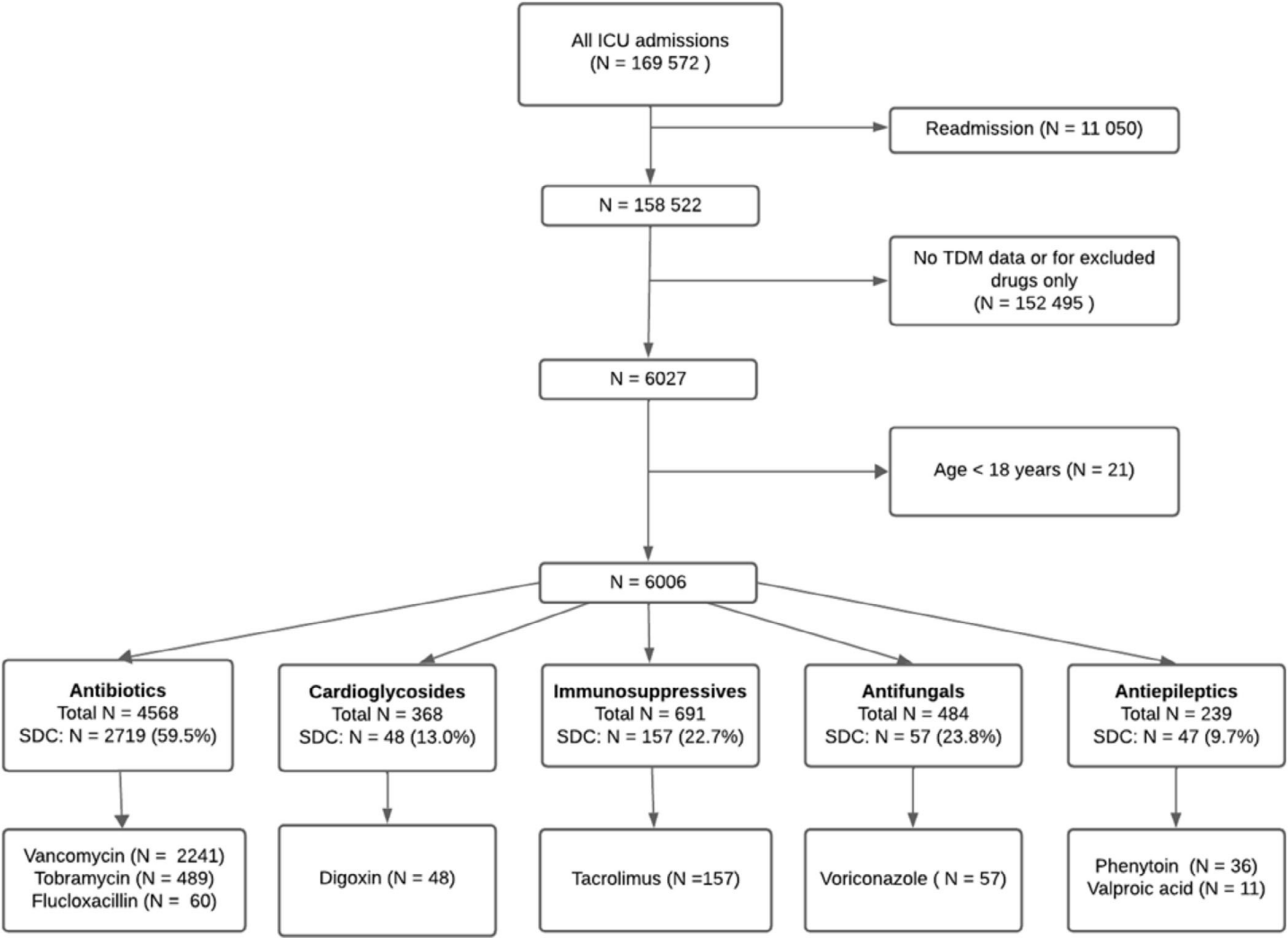
ICU admission inclusion flowchart with exclusion reasons and their counts. The counts per drug group indicate the number of ICU admissions with therapeutic drug monitoring measurement for one or more drugs in the drug group and the number of ICU admissions with at least one supratherapeutic drug concentrations (SDC) for one or more drugs in the specific drug group. The individual drug counts in the bottom panel indicate the number of ICU admissions with at least one SDC for the specific drug.

Our linked database consisted of 169 572 ICU admissions in total. After applying inclusion and exclusion criteria, a total of 6006 ICU admissions were included (Figure 1).

### 3.3 Characteristics of included ICU admissions

Baseline characteristics of ICU admissions with and without eSDCT are shown in Table 2. Of the 6006 ICU admissions, 2973 (49.5%) were classified as exposed to eSDCT. Overall, the exposed and unexposed groups were largely comparable, although infectious diseases and acute kidney injury at ICU admission were more common in the eSDCT group. Baseline characteristics stratified by group‐level eSDCT are provided in Supporting Information S6–S10. The antibiotics group was the largest, comprising 4568 ICU admissions undergoing TDM for one of the included antibiotics, of whom 2719 (59.5%) were classified as exposed to an antibiotic eSDCT.

**TABLE 2:** Characteristics of intensive care unit admissions with and without an electronic supratherapeutic drug concentration triggers.

Characteristic	eSDCT exposed admissions (*n* = 2973, 49.5%)	eSDCT not exposed admissions (*n* = 3033, 50.5%)
Age (years) median (Q1–Q3)	64.0 (54.0–71.0)	64.0 (54.0–72.0)
Male sex (no. (%))	1859 (62.5)	1997 (65.8)
Length (meter) median (Q1–Q3)	1.8 (1.7–1.8)	1.8 (1.7–1.8)
Weight (kilogram) median (Q1–Q3)	79.0 (68.0–90.0)	80.0 (69.0–90.0)
BMI median (Q1–Q3)	25.9 (22.9–29.4)	25.5 (22.8–29.0)
Planned admission (no. (%))	530 (17.8)	565 (18.6)
Admission type		
Medical (no. (%))	1778 (61.6)	1774 (62.1)
Emergency surgical (no. (%))	630 (21.8)	653 (22.8)
Elective surgical (no. (%))	477 (16.5)	431 (15.1)
APACHE IV admission diagnosis category		
Cardiovascular (no. (%))	1390 (46.9)	1262 (41.7)
Gastrointestinal (no. (%))	557 (18.8)	426 (14.1)
Respiratory (no. (%))	506 (17.1)	493 (16.3)
APACHE IV mortality probability median (Q1–Q3)	0.30 (0.20–0.60)	0.30 (0.10–0.50)
GCS low median (Q1–Q3)	15 (13–15)	15 (11–15)
Chronic diseases at ICU admission		
Chronic renal insufficiency (no. (%))	319 (10.7)	354 (11.7)
Chronic dialysis (no. (%))	96 (3.2)	95 (3.1)
Chronic cardiovascular insufficiency (no. (%))	230 (7.7)	252 (8.3)
Immunological insufficiency (no. (%))	670 (22.5)	648 (21.4)
Respiratory insufficiency (no. (%))	222 (7.5)	284 (9.4)
COPD (no. (%))	340 (11.4)	414 (13.6)
Acute conditions at ICU admission		
Confirmed infection (no. (%))	1271 (42.8)	1123 (37.0)
Sepsis–admission diagnosis (no. (%))	809 (27.2)	608 (20.0)
Dysrhythmia (no. (%))	272 (9.1)	334 (11.0)
CPR (no. (%))	184 (6.2)	240 (7.9)
Acute kidney injury (no. (%))	789 (26.5)	603 (19.9)
CVA (no. (%))	70 (2.4)	86 (2.8)
Mechanical ventilation within 24 h of admission (no. (%))	2326 (78.2)	2361 (77.8)
ICU Length of Stay median (Q1–Q3)	13.8 (5.6–28.1)	7.5 (3.2–15.7)
ICU mortality (no. (%))	909 (30.6)	616 (20.3)
Hospital mortality (no. (%))	1135 (38.2)	845 (27.9)

Abbreviations: APACHE, acute physiology and chronic health evaluation; BMI, body mass index; COPD, chronic obstructive pulmonary disease; CPR, cardiopulmonary resuscitation; CVA, cerebro vascular accident; eSDCT, electronic supratherapeutic drug concentration trigger; GCS, glascow coma scale; ICU, intensive care unit.

### 3.4 Associations between eSDCTs and ICU patient outcomes

**FIGURE 2:**
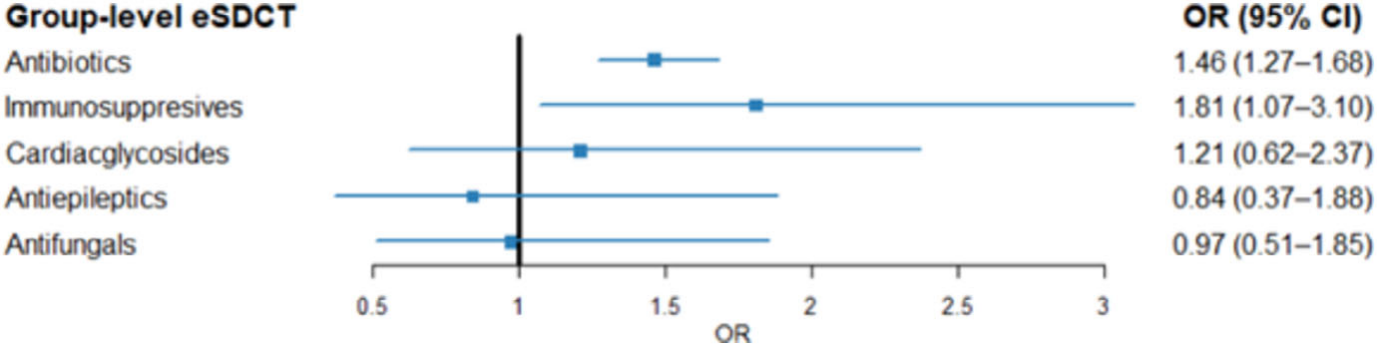
Forest plot showing odds ratios (OR) for group‐level electronic supratherapeutic drug concentration triggers (eSDCT) associated with hospital death. Error bars represent 95% confidence intervals (CI).

**FIGURE 3:**
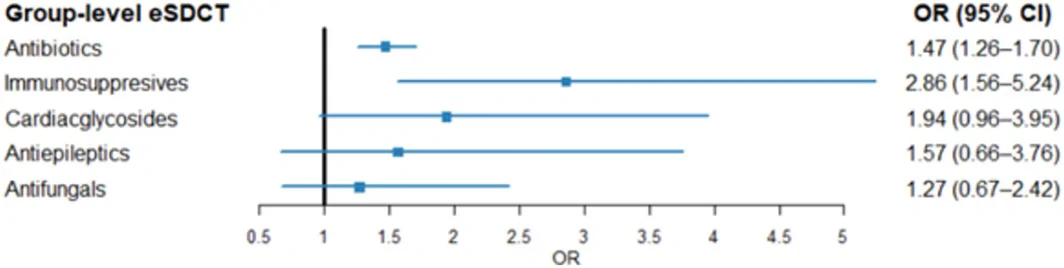
Forest plot showing odds ratios (OR) for with group‐level electronic supratherapeutic drug concentration triggers (eSDCT) associated with ICU death. Error bars represent 95% confidence intervals (CI).

**FIGURE 4:**
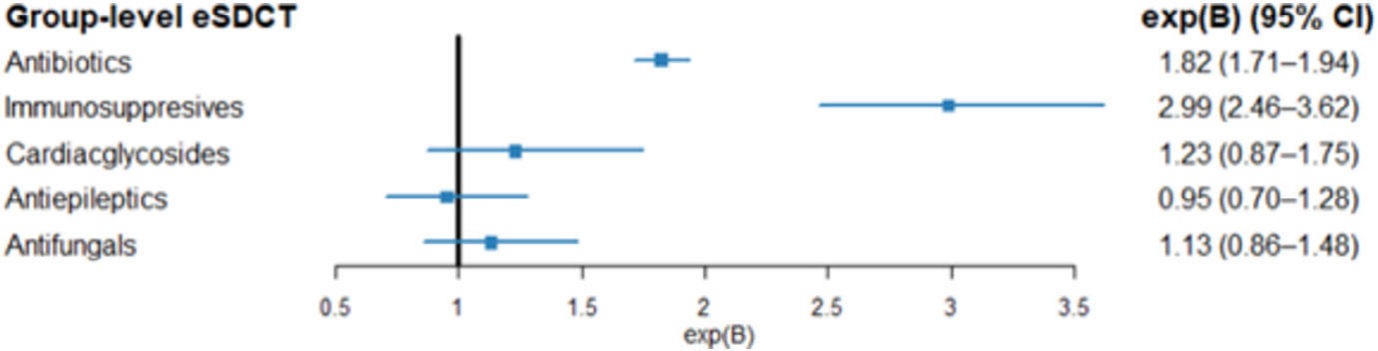
Forest plot showing Exponentiated coefficient (exp[B]) for group‐level electronic supratherapeutic drug concentration triggers (eSDCT) associated with ICU length of stay (LOS). Error bars represent 95% confidence intervals (CI).

Analyses were performed for the following five group‐level eSDCTs: antibiotics, antifungals, antiepileptics, cardiac glycosides and immunosuppressives. In the antibiotics group, eSDCT exposure was associated with a higher ICU and hospital mortality rate compared with no eSDCT exposure (Figures 2 and 3). The ICU LOS in the eSDCT exposed group was longer compared with the nonexposed group (Figure 4). The immunosuppressives cohort showed a similar pattern. The remaining cohorts showed no differences in outcomes between the eSDCT exposed and nonexposed group. The unadjusted results can be found in Supporting Information S11.

## 4 DISCUSSION

In this multicentre observational study, five group‐level eSDCTs pertaining to eight frequently administered drugs in ICU patients were investigated on their clinical value as an e‐trigger. By estimating associations between these eSDCTs and ICU patient outcomes rather than focusing on the PPV, we assessed their clinical value via a less labour‐intensive and data‐driven approach. In ICU patients exposed to antibiotic or immunosuppressive eSDCTs we found an association with worse ICU patient outcomes (longer ICU LOS and increased ICU and hospital mortality) compared with not exposed ICU patients, after adjusting for confounding.

Previous studies have demonstrated that each of the antibiotics included in the antibiotic eSDCT are associated with nephrotoxicity, particularly acute kidney injury (AKI), when present at supratherapeutic concentrations.^25^, ^26^, ^27^ Similarly, tacrolimus, the only drug included in the immunosuppressive eSDCT, is typically administered during clinically unstable phases, most notably in the early post‐transplantation period. These phases are characterized by fluctuating pharmacokinetics, which elevate the risk of tacrolimus‐induced nephrotoxicity.^28^, ^29^ AKI represents an adverse event with a relatively short time‐to‐onset following drug exposure and is therefore typically observable during ICU admission. This had important implications for ICU care.^30^ Considering the well‐documented association between AKI and adverse outcomes in critically ill patients, the observed associations for antibiotic and immunosuppressive eSDCTs with ICU patient outcomes align with the clinical evidence and may, at least in part, be attributable to AKI.^31^

Although hepatotoxicity has been linked to increased mortality and associated with supratherapeutic voriconazole concentrations (the only drug included in the antifungal eSDCT), we did not observe an association between antifungal eSDCT and ICU patient outcomes.^32^, ^33^, ^34^ This discrepancy may partly reflect inconsistent evidence for the clinical consequences of supratherapeutic voriconazole levels. In particular, the upper therapeutic threshold remains debated, with variable associations reported for hepatotoxicity.^35^, ^36^, ^37^, ^38^ Furthermore, the time‐to‐onset of hepatotoxicity can range from days to months, often extending beyond typical ICU stays.^37^, ^38^, ^39^ This suggests that ICU data alone may be insufficient to fully capture the clinical impact of supratherapeutic voriconazole concentrations. Similarly, for digoxin (the only drug included in the cardiac glycoside eSDCT) the clinical effects of supratherapeutic concentrations have shown considerable variability across studies.^40^, ^41^, ^42^, ^43^, ^44^ This complicates the establishment of a definitive threshold and potentially explains the lack of observed association with ICU patient outcomes in our study. Lastly, no association was found between antiepileptic eSDCTs and clinical outcomes. Studies show that severe adverse effects from antiepileptic drugs typically occur only at markedly elevated levels. Slight elevations above the therapeutic range are generally not linked to increased mortality or prolonged ICU LOS and are often reversible.^45^, ^46^ Thus, for antiepileptic drugs, the lack of observed association may similarly reflect the chosen threshold for the corresponding eSDCT.

### 4.1 Strengths and limitations

This study has several strengths. To the best of our knowledge, this is the first multicentre study on the associations between eSDCTs and ICU patient outcomes, reusing routinely collected pseudonymized EHR data. Our study provides a new perspective on the assessment of the clinical utility of ADE e‐triggers before implementing them for medication safety surveillance. Second, we adjusted for a wide range of potential confounding factors, which provides a more accurate estimate of the associations. Third, the eSDCTs were identified in three consecutive steps to ensure their relevance for clinical practice and their thresholds were determined via nationally prevailing online TDM resources.

This study has several limitations that should be considered when interpreting the results. An important limitation of ICU LOS as an outcome is that short stays may reflect either rapid recovery or early death, which complicates the interpretation of the effect estimates. Therefore, ICU LOS should be interpreted in conjunction with other study outcomes. Additionally, due to limited data availability, the immunosuppressives and cardiac glycoside models had an Events Per Variable (EPV) below 10, 8.9 and 8.4 respectively. However, in these cases, we prioritized adequate control for confounding over strict adherence to the conventional EPV ≥ 10 rule, as proposed in clinical epidemiology literature.^47^, ^48^

Furthermore, the defined thresholds were applied as absolute cut‐off points. We did not consider the extent to which these thresholds were exceeded, nor did we account for potential dose adjustments following the detection of supratherapeutic concentrations. The former may have influenced the observed associations in either direction, while the latter could have attenuated them. However, as mentioned earlier, the cut‐offs were chosen based on nationally prevailing online resources which contain evidence‐based TDM guidelines. Furthermore, our data contained trough and peak TDM measurements based on documented sampling times in EHRs. As this was a retrospective study using routinely collected data, we were unable to verify whether the documented sampling times fully correspond to the actual blood draw times. In routine clinical practice, discrepancies between actual and documented sampling times may occur. Such potential misclassification could have influenced drug level categorization and thereby impacted our results.^49^, ^50^ Similarly, our data originated from 14 ICUs in the Netherlands. ICUs may differ in the timeliness and type of actions taken after identifying supratherapeutic drug concentrations. Consequently, some ICUs may implement faster or more effective measures than others, potentially attenuating the observed effects on patient outcomes. Furthermore, the first TDM measurement is often used to guide subsequent dosing and adjust if needed based on the concentration found. Consequently, this initial measurement may more frequently reflect extreme or non‐steady‐state concentrations compared with later measurements.^45^, ^51^ However, such early deviations in drug exposure may already predispose ICU patients to toxic effects, underlining the need to account for this critical phase when investigating the associations between eSDCTs and patient outcomes.

Lastly, as in any observational study, we only adjusted for confounding factors that were measured and recorded. It cannot be excluded that some unmeasured confounding factors may have affected our results.

### 4.2 Implications for clinical practice and future studies

We demonstrated that ICU patients flagged by specific eSDCTs had a higher likelihood of mortality and a longer ICU LOS. Although these findings do not establish causality, they indicate that the identified eSDCTs are associated with clinically meaningful patient outcomes. These associations suggest that implementing eSDCTs on ICU‐level dashboard may help to identify areas for medication safety improvement. Therefore, they can serve as a quantitative basis for clinicians and healthcare organizations to assess TDM and drug dosing processes and aim for improvements in for example clinical pharmacy services in the ICU.^52^

In this regard, the proposed data‐driven methodology allows for a more efficient evaluation of the clinical utility of e‐triggers compared with the traditional, labour‐intensive manual chart review by medical experts to assess the PPV for each individual trigger. Focusing exclusively on e‐triggers linked to adverse patient outcomes enables a more strategic and efficient selection process, facilitating earlier validation, enhancing uptake of e‐triggers for ICU‐level medication safety surveillance. As such, it provides a basis for refinement of eSDCTs and development and validation of other ADE e‐triggers across different ICU settings. Findings from such surveillance can subsequently be used to target medication safety interventions.

Furthermore, in recent years, there has been a notable increase in the availability of EHR data, suggesting that more detailed information may now be accessible than at the time of earlier studies.^53^, ^54^ Such data could potentially be leveraged to further refine ADE e‐triggers, such as eSDCTs. Future studies could then apply the proposed methodology to assess whether these modifications strengthen the association between eSDCTs and patient outcomes. For example, studies could focus on eSDCTs representing individual drugs rather than entire drug classes, provided that the number of cases is sufficient.^55^ Additionally, existing evidence suggests that some thresholds may be suboptimal, which could weaken the observed associations between eSDCTs and ICU patient outcomes. Investigation of alternative thresholds for individual eSDCTs may therefore provide further insights. Other improvements could include incorporating multiple rules, such as patient characteristics and biochemical markers, and accounting for specific populations (like patients on extracorporeal membrane oxygenation, who exhibit high pharmacokinetic variability) while also considering TDM patterns over time.^9^, ^14^, ^56^, ^57^ Lastly, besides eSDCTs, there are more types of e‐triggers that are and can be studied using our approach, such as triggers derived from subtherapeutic drug concentrations, biochemical abnormalities and antidote administration.^4^, ^23^, ^58^

## 5 CONCLUSION

By estimating the associations between eSDCTs and ICU patient outcomes and leveraging routinely collected EHR data, we evaluated the clinical utility of five group‐level eSDCTs for medication safety monitoring. Our data‐driven approach provides a resource‐efficient alternative to conventional manual validation methods, enabling faster clinical utility assessment of e‐triggers such as eSDCTs. This may enhance their implementation for ICU‐level medication safety surveillance. Given the observed association between antibiotic and immunosuppressive eSDCTs and worse ICU patient outcomes, these triggers show potential for such implementation. For the remaining eSDCTs, additional research is needed with a focus on identifying best‐suited thresholds for detecting potential ADEs.

## THE RESCUE STUDY GROUP MEMBERS

Bouman, C.S.C.^4^; van Roon, E.N^5^.; Schutte, P.F.^6^; ten Cate, J.^6^; van Balen, D.^7^; Hendriks, S.^8^; Lau, C.^9^; Vermeijden, W.J.^10^; Beishuizen, A.^10^; Masselink, J.B.^11^; Spronk, P.E.^12^; van Kan, H.J.M.^13^; de Feiter, P.W.^4^; Wils, E.J.^14^; Wieringa, A.^15^; Valkenburg, A.J^16^; van den Bergh, W.M.^17^; Renes, M.H.^17^; Bult, W.^17,18^; de Jonge, E^19^.; Hoeksema, M.^20^; Wesselink, E.J^21^; Purmer, I.M.^22^; Bosma, B.E.^23^; van Bree, S.H.W.^24^; Lammers, H.J.W^25^; Bosman, R.J.^26^; Franssen, E.J.F.^27^; Karakus, A.^28^; Sigtermans, M.^28^; Kuck, E.M.^29^; Dongelmans, D.^A4^; Aries, M.J.H^30,31^; Uijtendaal, E.V^32^; Reuland, M.^C4^;

^4^Amsterdam UMC location University of Amsterdam, Department of Intensive Care Medicine, Meibergdreef 9, Amsterdam, The Netherlands

^5^Department of Clinical Pharmacy and Pharmacology, Frisius medical Center, Leeuwarden, The Netherlands

^6^Department of Intensive Care, The Netherlands Cancer Institute, Amsterdam, The Netherlands

^7^Department of Pharmacy & Pharmacology, The Netherlands Cancer Institute, Amsterdam, The Netherlands

^8^Department of Intensive Care, Albert Schweitzer Ziekenhuis, Dordrecht, The Netherlands

^9^Department of Hospital Pharmacy, Albert Schweitzer Ziekenhuis, Dordrecht, The Netherlands

^10^Department of Intensive Care, Medisch Spectrum Twente, Enschede, The Netherlands

^11^Department of Clinical Pharmacy, Medisch Spectrum Twente, Enschede, The Netherlands

^12^Department of Intensive Care Medicine, Gelre Hospitals, Apeldoorn, The Netherlands

^13^Department of Clinical Pharmacy, Gelre Hospitals, Apeldoorn, The Netherlands

^14^Department of Intensive Care, Franciscus Gasthuis & Vlietland, Rotterdam, The Netherlands

^15^Department of Clinical Pharmacy, Isala Hospital, Zwolle, The Netherlands

^16^Department of Intensive Care, Isala Hospital, Zwolle, The Netherlands

^17^Department of Critical Care, University Medical Center Groningen, University of Groningen, Groningen, The Netherlands

^18^Department of Clinical Pharmacy and Pharmacology, University Medical Center Groningen, University of Groningen, Groningen, The Netherlands

^19^Department of Intensive Care, Leiden University Medical Center, Leiden, The Netherlands

^20^Department of Anesthesiology, Intensive Care and Painmanagement, Zaans Medisch Centrum, Zaandam, The Netherlands

^21^Department of Clinical Pharmacy, Zaans Medisch Centrum, Zaandam, The Netherlands

^22^Department of Intensive Care, Haga Hospital, The Hague, The Netherlands

^23^Department of Hospital Pharmacy, Haga Hospital, The Hague, The Netherlands

^24^Department of Intensive Care, Ziekenhuis Gelderse Vallei, Ede, The Netherlands

^25^Department of Hospital Pharmacy, Ziekenhuis Gelderse Vallei, Ede, The Netherlands

^26^Department of Intensive Care, Onze Lieve Vrouwe Gasthuis, Amsterdam, The Netherlands

^27^Department of Clinical Pharmacy, Onze Lieve Vrouwe Gasthuis, Amsterdam, The Netherlands

^28^Department of Intensive Care, Diakonessenhuis Utrecht, Utrecht, The Netherlands

^29^Department of Hospital Pharmacy, Diakonessenhuis Utrecht, Utrecht, The Netherlands

^30^Department of Intensive Care, Maastricht University Medical Center, Maastricht, The Netherlands

^31^Mental Health and Neurosciences Institute, University Maastricht, Maastricht, The Netherlands

^32^Department of Clinical Pharmacy, University Medical Center Utrecht and Utrecht University, Utrecht, The Netherlands.

## AUTHOR CONTRIBUTIONS

**Anne P. Langermans**: Conceptualization; methodology; formal analysis; investigation; writing—original draft. **Izak A.R. Yasrebi‐de Kom**: Conceptualization; data curation; methodology; formal analysis; validation; writing—review and editing. **Dylan W. de Lange**: Conceptualization; funding acquisition; writing—review and editing. **Nicolette F de Keizer**: Conceptualization; data curation; methodology; validation; writing—review and editing; supervision; project administration; resources; funding acquisition. **Joanna E. Klopotowska**: Conceptualization; data curation; methodology; validation; writing—review and editing; supervision; resources; funding acquisition. All authors and collaborators approved the submitted manuscript.

## CONFLICT OF INTEREST STATEMENT

APL is a PhD candidate at Amsterdam University Medical Center (UMC) and is part‐time employed by ChipSoft, Amsterdam. The employment at ChipSoft is unrelated to any aspect of the present study. NdK and DdL are members of the board of the NICE registry. The department of Medical Informatics, where AL, IdKY, JK and NdK are employed, receives funding to process data of Dutch ICUs into the NICE registry. There are no other conflicts of interest.

## Supporting information


**Data S1.** Supporting Information.

## Data Availability

The datasets collected and analysed in this study are not publicly available due to the data sharing agreements with the participating ICUs. The access to the data might only be provided after explicit consent from each separate participating ICU.
